# Real‐time CO_2_ monitoring for early detection of grain spoilage and mycotoxin contamination

**DOI:** 10.1002/jsfa.70151

**Published:** 2025-08-28

**Authors:** Elisavet Kiaitsi, Sandro Zanardi, Michael Sulyok, Angel Medina, Ben Ingram, Michele Suman, Esther Garcia‐Cela

**Affiliations:** ^1^ School of Cardiovascular and Metabolic Medicine & Sciences King's College London London UK; ^2^ Sensory and Analytical Food Science Barilla G e R Fratelli SpA Parma Italy; ^3^ Institute for Bioanalytics and Agrometabolomics, Department of Agrobiotechnology (IFA‐Tulln) University of Natural Resources and Life Sciences Vienna Tulln Austria; ^4^ Magan Centre of Applied Mycology Cranfield University Cranfield UK; ^5^ Department of Interactive Visualization and Virtual Reality, Faculty of Engineering Universidad de Talca Talca Chile; ^6^ Department for Sustainable Food Process Catholic University Sacred Heart Piacenza Italy; ^7^ MycoLab, Clinical, Pharmaceutical and Biological Sciences, School of Health, Medicine and Life Sciences, Centre for Agriculture, Food and Environmental Management Research University of Hertfordshire Hatfield UK

**Keywords:** wheat, silo, fungi, relative humidity, cereals, food waste

## Abstract

**BACKGROUND:**

This study aimed to compare the use of real‐time CO_2_ , temperature (*T*) and relative humidity (RH) sensors as indicators of stored grain quality management, specifically for early detection of mould activity and mycotoxin contamination. Initial experiments were conducted using mini‐silos containing naturally contaminated wheat grain (1.5 kg) stored at different moisture contents of 15–30% (water activity, *a*_w_ = 0.78 to 0.98), to evaluate their effects on grain respiration.

**RESULTS:**

Respiration rates and dry matter losses increased with grain moisture content. A larger‐scale, nine‐month study was then conducted using two pilot‐scale silos (2.5 t; 1400 cm diameter; 2050 cm height) equipped with ATEX‐compliant CO_2_/RH/*T* sensors. A ‘wet pocket’ was simulated by introducing water to a localised area to mimic a water ingress event. This led to a rapid rise in CO_2_ levels while *T* remained relatively stable. Mycotoxin analyses of the affected and unaffected regions showed a clear increase in the concentration and diversity of mycotoxins, particularly aflatoxin B1, aflatoxin B2, deoxynivalenol, deoxynivaenol‐3‐glucoside and moniliformin, in the wet pocket area.

**CONCLUSION:**

Real‐time CO_2_ monitoring provided a faster and more sensitive indication of spoilage and mycotoxin risk compared to *T* and RH measurements. This highlights the potential for developing early‐warning systems for stored grain management based predominantly on continuous CO_2_ monitoring. © 2025 The Author(s). *Journal of the Science of Food and Agriculture* published by John Wiley & Sons Ltd on behalf of Society of Chemical Industry.

## INTRODUCTION

Cereals, especially wheat, maize and rice, are staple food commodities of global importance from both food security and economic points of view. Moreover, they are often contaminated with mycotoxins, which are secondary metabolites produced mainly by *Aspergillus*, *Penicillium* and *Fusarium* species.^1^, ^2^ Mycotoxins are heat‐stable molecules, difficult to remove or inactivate once formed in a food product. Exposure mainly by ingestion can result in immunotoxicity, teratogenicity, mutagenicity and carcinogenicity.^3^ Consequently, there are international regulations to protect consumers. Notably, in the European Union/UK, there is a maximum legal limit in cereals for aflatoxins, ochratoxin A, deoxynivalenol, zearalenone, T2 and HT2.^4^ However, the lack of harmonisation in legal limits compromises global market accessibility.^5^ Cereals are often colonised by fungal species in the field; therefore harvested cereals often need to be effectively dried prior to on‐farm storage or in more centralised silo systems. Thus, today in the framework of the Hazard Analysis and Critical Control Point (HACCP) for cereal chains to prevent mould/pest spoilage and mycotoxin contamination, key critical control points including drying to safe moisture contents (m.c.) and postharvest storage management have been identified.^6^

Postharvest storage of cereals is thus a critical and key phase in the cereal food/feed chain because poor management and monitoring can result in significant quality losses, due to either pest activity or mould spoilage and associated mycotoxin contamination.^7^, ^8^ Poor postharvest drying and storage can result in significant losses and mycotoxin contamination, quantified as 5–10% in developed countries and up to 30–35% in lower middle‐income countries.^9^ It is important that effective monitoring and control systems can be utilised to minimise the impact of pests, spoilage moulds and mycotoxins which may affect grain quality and to facilitate the development of appropriate storage management solutions to conserve quality.

The challenge of postharvest grain management is further compounded by climate change impacts. Kerry *et al*.^10^ demonstrated that future climate scenarios predict increased aflatoxin contamination risk in major grain‐producing regions, highlighting the urgent need for improved monitoring technologies. This emphasises the importance of developing robust, real‐time detection systems that can adapt to changing environmental pressures on stored grain quality.

Staple cereal commodities are usually alive and respiring. The actual level of respiration of the grain and the associated mycobiota is influenced by the prevailing temperature (*T*) and m.c. when entering the storage phase and by the type of facility. The most important abiotic factors in storage systems are the prevailing temperature upon storage entry, the inter‐granular atmosphere (CO_2_ level) and the relative humidity (RH).^7^, ^8^, ^11^

The *T* and indeed RH in the inter‐granular spaces in grain silos have been previously measured intermittently or by using sensor networks. For example, *T* sensor networks have been developed to place in a detailed matrix configuration within grain silos to monitor changes in real time as a monitoring system for managing stored grains silos.^12^ Indeed, this approach using *T* sensors with wireless connectivity has now become quite commonplace in grain silos usually as steel tethered cables in cereal silos. However, as grain is a very good insulator the question arises as to whether *T* reflects earlier respiratory activity (CO_2_), and whether the latter may be a more sensitive indicator of initiation of mould spoilage or pest activity in stored cereals.

Studies at Purdue University examined the potential use of CO_2_ as an indicator of the activity of spoilage moulds and perhaps pests.^13^ They suggested that CO_2_ monitoring was a sensitive method for detecting hotspots due to water ingress at an earlier stage than other available methods of detection.^14^ Their approach was originally to place sensors in the head‐space and/or exhaust outlet fans. However, the question arises as to whether by measuring CO_2_ the increased sensitivity and early indication of biological activity in stored cereals occurs prior to changes in *T*. Since cereals are good insulators, the presence of pockets of wet grain would likely be detected at a much later stage if only using temperature changes as an indicator, whereas changes in CO_2_ concentration levels due to more rapid diffusion through the grain pore space are more likely to be detected at an earlier stage. The use of CO_2_ as an indicator of stored grain quality management is gaining interest in the scientific community.^14^ In fact, recent studies have evaluated the use of this approach not only for monitoring stored cereals but also for transportation.^15^, ^16^ However, there have been few studies of the correlation between CO_2_ and mycotoxin production.^17^

The relationship between CO_2_ production in stored cereals under different *T* × m.c. storage conditions has been studied in detail in both temperate and tropical cereals and peanuts. The temporal and total cumulated CO_2_ production has been used to quantify the relative dry matter losses (DMLs) under different safe, intermediate and unsafe storage conditions for mould spoilage.^18^, ^19^, ^20^, ^21^, ^22^, ^23^, ^24^, ^25^ However, these studies were performed on a small scale and the challenge remains for large‐scale grain storage. In addition, the DMLs under different *T* × m.c. conditions have been used to correlate with the levels of mycotoxins present under different storage conditions. This has the advantage of identifying the DML tolerances with regard to meeting the prevailing legislative limits for specific mycotoxins and prevention of exceeding these limits during storage. DML was used as a grain quality indicator with values as low as 0.04% DML being considered to have an impact on seed germination and early visible fungal presence on wheat, while DML of >1% and between 0.5% and 0.75% would represent a high risk of zearalenone and deoxynivalenol levels above the EU/UK legislative limits respectively.^18^, ^19^ Mylona *et al*.^25^ showed that a 0.5% DML in stored maize was enough to downgrade this commodity from food to feed grade. In addition, DMLs of <1% resulted in maize contaminated with *F. verticillioides* exceeding the EU legislative limits for fumonisins as well as in maize contaminated with *A. flavus* exceeding aflatoxin B1.^20^, ^25^

Recent advances in predictive modelling have further enhanced the potential for proactive grain management. Ingram *et al*.^26^ developed *a*_w_/*T* models that can predict fungal growth and mycotoxin production under specific storage conditions. Such models, when integrated with real‐time CO_2_ monitoring systems as demonstrated in this study, could provide a comprehensive decision support framework for silo managers to predict contamination risk before visible spoilage occurs.

Hence, there is interest in the development of more proactive systems for monitoring of stored cereals to improve postharvest management. The objectives of the study reported here were to: (a) examine and monitor the real‐time changes in both CO_2_ and temperature in wheat grain stored at different m.c. and (b) evaluate of CO_2_, *T* and RH sensors suspended from integrated sensor cables (3 × 4) in a pilot‐scale silo, where wheat was stored, in real time for a period of 9 months and introduction of water in a specific region of the pilot silo to examine effects on abiotic parameters. The results are discussed in the context of the development of a decision support system.

## MATERIALS AND METHODS

### Sensor systems

Two sensor systems were used in this study. For the laboratory mini‐silo experiments, a custom setup was assembled consisting of a RH sensor and an infrared CO₂/*T* sensor (0–15% ±0.2% and 0–45 °C ±0.25 °C respectively) (Analox, UK). Data were automatically recorded on a digital control system and then downloaded into an Excel file for handling and analysis. The second system, developed and supplied by Gescaser SA (Almacelles, Lleida, Spain), was an integrated platform designed for larger‐scale silo monitoring. It included sensors for CO₂ (0–10 000 ppm), temperature (−25 to 60 °C) and moisture (0–100%). This system was connected to CTC+ v3.5.4.0 desktop software, which enabled real‐time monitoring and visualisation of grain, silo air and ambient environmental conditions through a graphical user interface.

#### Mini‐silo experiments

An adsorption curve for winter wheat (Camgrain, UK)^19^ was used for this experiment. Different amounts of water were added to 2.5 kg aliquots of wheat grain, which were then stored at 4 °C in sealed containers and shaken regularly to equilibrate the grain at target m.c. values. Final m.c. and water activity (*a*_w_) values were checked and confirmed by oven drying (115 °C overnight) a subsample of the winter wheat, by measuring *a*_w_ with a water activity meter (Aqualab TE4, Meter Group, USA). The m.c. values were confirmed as 15%, 16.5%, 18.0%, 21.5%, 25%, >27.5% (*a*_w_ = 0.70, 0.80, 0.85, 0.90, 0.95 and 0.98). Subsequently, 1.5 kg wheat samples with different initial m.c. were placed in individual thermos flasks equipped with the aforementioned Analox sensors. The grain was then incubated at 25 °C for up to 35 days. All the mini‐silo experiments were carried out in duplicate. Cumulative respiration and DMLs were calculated as described elsewhere.^19^

**Figure 1:**
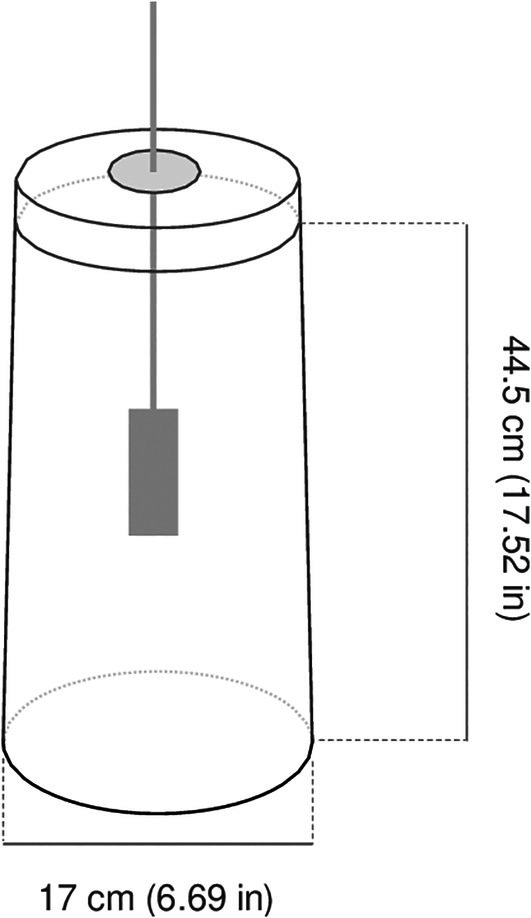
Schematic of the mini‐silo experiment. Wheat samples (1.5 kg) adjusted to six water activity levels (*a*_w_ = 0.70, 0.80, 0.85, 0.90, 0.95 and 0.98) were incubated in sealed thermos flasks (diameter 17 cm, height 44.5 cm) equipped with Analox sensors at 25 °C for up to 35 days. All experiments were conducted in duplicate.

For these experiments we used Analox sensors. The sensors were positioned centrally inside 5 L thermos flasks (Thermo, UK) (Fig. 1). Each container featured a detachable lid and with detachable lids and a double layer of cotton wool placed on top to close the system.

#### Pilot‐scale silo experiments

**Figure 2:**
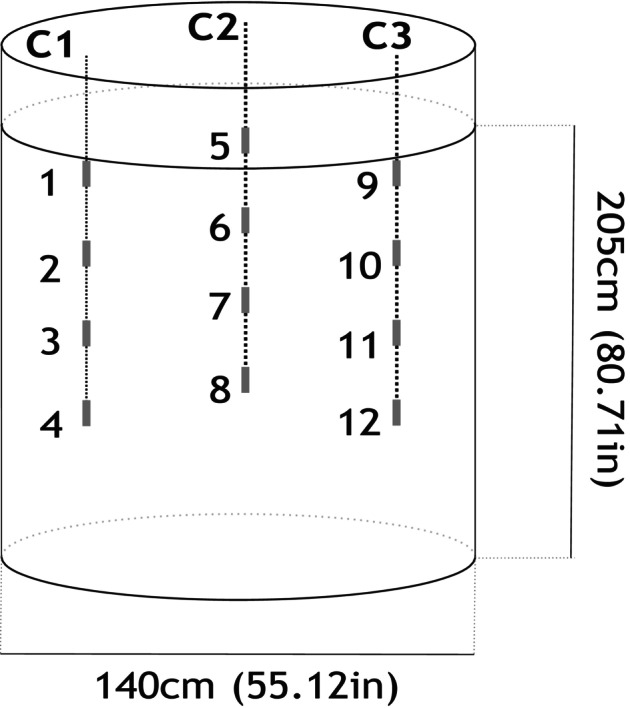
Schematic of the pilot‐scale silo experiment. Two pilot silos (diameter 1.4 m, height 2.05 m), each filled with *ca* 2.5 t of durum wheat (14% m.c.), were installed at Barilla SpA (Parma, Italy) on 8 August 2017. One silo was placed indoors and the other outdoors under ambient conditions. Each silo was equipped with three sensor cables (vertical dotted lines labelled C1–C3), each containing four sensor nodes (temperature, RH and CO_2_) labelled 1–12, spaced *ca* 1.5 m apart and positioned equidistantly within the grain mass.

Pilot‐scale silo experiments were performed at Barilla SPA, Parma, Italy facilities. On 8 August 2017, two pilot silos (1400 cm diameter and 2050 cm height) were filled with approximately 2.5 t of durum wheat (14% m.c.) destined for pasta production. One silo was located inside the facility, while the other was placed outside under ambient conditions. Three cables composed of four integrated sensor nodes (*T*/RH and CO_2_ each; approx. 1.5 m distance between them) were tethered inside the silo at an equidistant separation from each other (Fig. 2). The power source for the system was designed to minimise total power consumption so as to minimise the heat generated during the measurements, ensuring that the sensor was only switched on briefly while taking the required measurements. The electronic design was also integrated with a wireless module that allowed the transmission of the measured signals to a remote‐control module either via wireless or by cable. The multiplexer board was automatically switched between the sensor nodes in each sequence, recording data on current CO_2_, *T* and RH. The data were downloaded on a weekly and monthly basis, loaded into Excel and analysed for trends and changes in abiotic factors. Measurements were taken every 30 min and data were recorded over a period of 9 months (October 2017–May 2018). On 4 May 2018, a wet ‘hotspot’ was created in the silo located outside the facilities, increasing the m.c. of the wheat from 12.5% to 18% in the area surrounding sensor 9, attached to cable 3.

### Mycotoxin analysis

Mycotoxin analysis was conducted exclusively on samples from the pilot‐scale experiment, focusing on the silo in which the hotspot was induced. Two aggregate samples were collected for analysis: one from the artificially created hotspot area and one from regions located far from the hotspot, where CO₂ levels remained near baseline. Each aggregate sample was obtained from the homogenisation of a total mass of 1 kg obtained by combining 10 samples of 100 g each at the height and in the area of the silo zone of interest.

Samples were frozen immediately after collection, and the experiment was carried out 6 days after the water simulation. Grain was dried at 60 °C for 48 h, milled and stored at 4 °C until analysis. An amount of 5 g of each milled sample was extracted using 20 mL of extraction solvent (acetonitrile–water–acetic acid, 79:20:1, v/v/v) followed by a 1 + 1 dilution using acetonitrile–water–acetic acid (20:79:1, v/v/v) and direct injection of 5 μL of diluted extract into the sampling port for liquid chromatography–tandem mass spectrometry analysis as described elsewhere.^27^ The limit of quantification and limit of detection (LOD) in μg kg^−1^ were as follows: aflatoxin B1 (0.05/0.16), aflatoxin B2 (0.06/0.19), ochratoxin A (0.08/0.28), deoxynivalenol (1.2/3.9), moniliformin (0.900/3.0).

### Statistical analysis

Statistical analyses were performed using R statistical software (version 4.3.0). Data normality was assessed using the Shapiro–Wilk test. For non‐normally distributed data, non‐parametric tests were employed.

To evaluate the relationship between CO_2_ levels and mycotoxin contamination, sensor nodes were classified into two groups based on measured mycotoxin detection levels. The low CO_2_ nodes (nodes where mycotoxins were below LOD) and high CO_2_ node (nodes in regions where levels of mycotoxins were detected). A Mann–Whitney *U* test was conducted to compare CO_2_ concentrations between these groups using all CO_2_ measurements from the respective nodes across the monitoring period.

Temporal analysis was performed to assess changes in environmental parameters before and after the water addition event on 14 May 2018. Data were divided into pre‐water (August 2017–April 2018) and post‐water (May 2018) periods. Wilcoxon signed‐rank tests were used to compare CO_2_, temperature and RH levels between these periods.

Given the limited number of sensor nodes of data available, descriptive analysis was used to examine relationships between CO_2_ levels and mycotoxin presence, with particular focus on threshold identification for early‐warning applications. Statistical significance was set at *P* < 0.05 for all analyses.

## RESULTS

### Initial studies on CO_2_ changes in wheat stored at different constant m.c. × *T* conditions in mini‐silos

**Figure 3:**
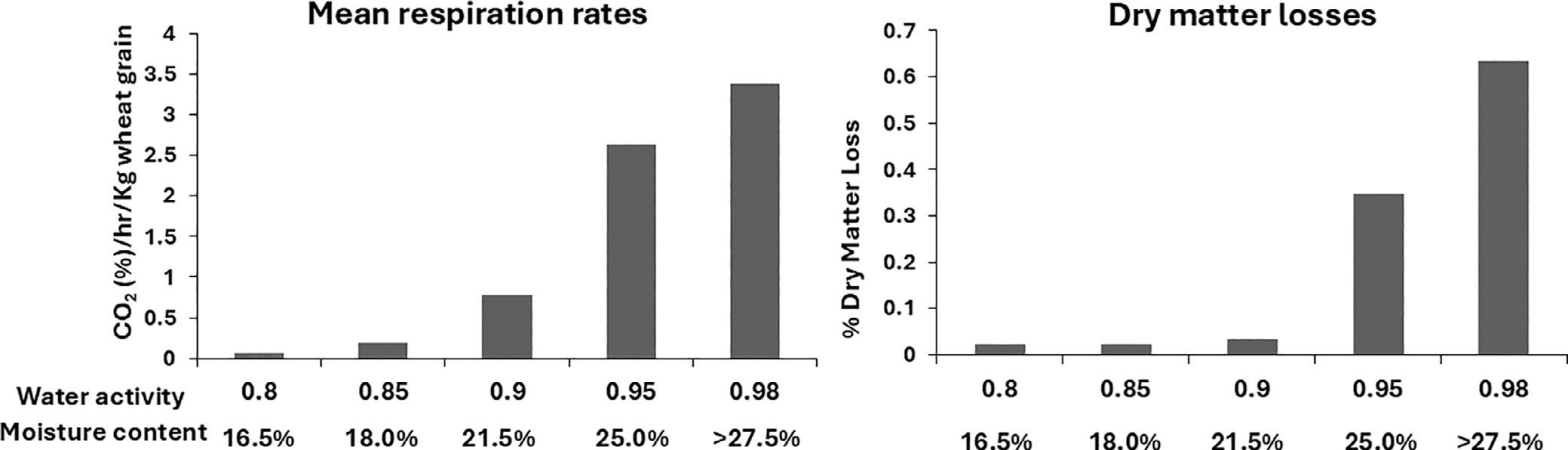
Natural wheat respiration at different m.c. stored at 25 °C for 7 days. Average of two replicates.

Respiration of natural wheat stored at 25 °C was monitored for 5 days (Fig. 3). The production of CO_2_ clearly increases with an increase of m.c, particularly from humidity levels of 21.5% and above. Furthermore, DMLs higher than 0.3% can be observed at 25% m.c.

### Studies on relationship between CO_2_ and *T* changes in wheat under intermediate constant m.c. conditions

**Figure 4:**
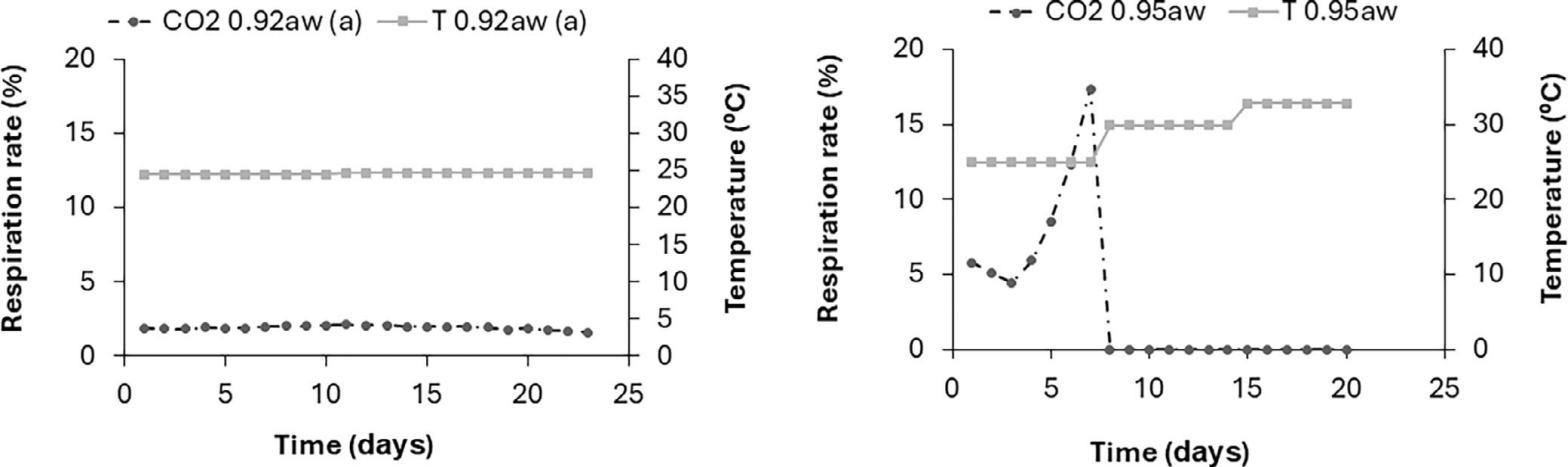
*T* and CO_2_ temporal changes for natural wheat respiration at *a*_w_ = 0.92 (22% m.c) and *a*_w_ = 0.95 (25% m.c) stored at 25 °C for 25 days.

Figure 4 depicts the respiration rate and the temperature of 1.5 kg of natural wheat for 25 days at 25 °C and different m.c. regimens. At 22% m.c. (*a*_w_ = 0.92), *T* did not increase during the entire period of storage. In contrast, an increase in the respiration rate was observed after 5 days of incubation, reaching a maximum (2.2%) at day 10. Later, respiration rate decreased due to grain desiccation. Increases were observed in natural wheat at 25% m.c not only with respiration rate but also in *T*. However, increase of CO_2_ production was faster than increases in *T*. The CO_2_ sensor maximum detection level was reached at day seven at *a*_w_ = 0.95 (17.5% m.c). Similar results were observed in the other conditions tested; for example, at 31% m.c., CO_2_ started increasing quickly after 2 days of storage at 20 °C, and after 7–8 days at 15 °C. In both cases the temperature showed an increase later in the process, or no increase at all (data not shown).

### Pilot‐scale silo experiments

**Figure 5:**
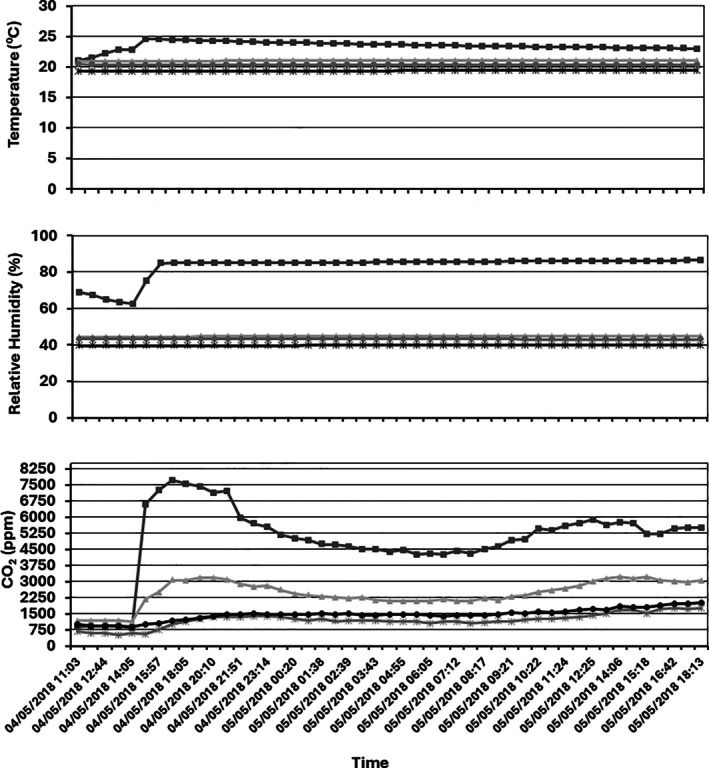
*T*/RH/CO_2_ reading from the four nodes in cable 3 after increasing the m.c. in node 9 (blue line) on 5 April 2018. Blue line, node 9; orange line, node 10; yellow line, node 11; grey line, node 12 within an outside pilot silo at Barilla (Italy).

In order to monitor the environmental conditions, two sensors were located next to each pilot silo. RH and *T* fluctuated throughout the 9 months of storage time (Fig. SA1). Environmental conditions ranged between 10–30 °C/27.5–57% RH and 0–35 °C/30.5–92.0% RH inside and outside the facility respectively. Erroneous readings from the sensor were occasionally received for the RH measurements. Values recorded during an eight‐month period by the four nodes in one of the three cables showed that initial measures on the three variables recorded demonstrated high variability (first month) and therefore a period of equilibration is required before considering accurate measurements can be made (Figs. SA2 and SA3). In addition, sporadically some erroneous values are observed. However, after equilibration, values observed in the four different levels across the silo were homogeneous as expected (same RH of around 40%). Similarly, as was observed with the sensors outside the silo, temperature was the variable that presented higher fluctuations (*ca* 20 °C fluctuation outside and 10 °C fluctuation outside). Humidity and CO_2_ values were less susceptible to environmental changes and therefore are robust parameters to consider. On 4 May 2018 a wet ‘hotspot’ was created in the silo located outside the facilities increasing the m.c. of the wheat from 12.5% to 18% in the area of sensor 9 attached to cable 3 (Fig. 2). The impact on *T*/HR/CO_2_ production in the four nodes (9, 10, 11 and 12) is presented in Fig. 5. Although high increases in all variables were observed, the CO_2_ increases was substantially higher. Moreover, contrary to what was seen in *T* and HR levels, changes in the CO_2_ production were also recorded in the rest of the nodes attached to the same cable.

**Figure 6:**
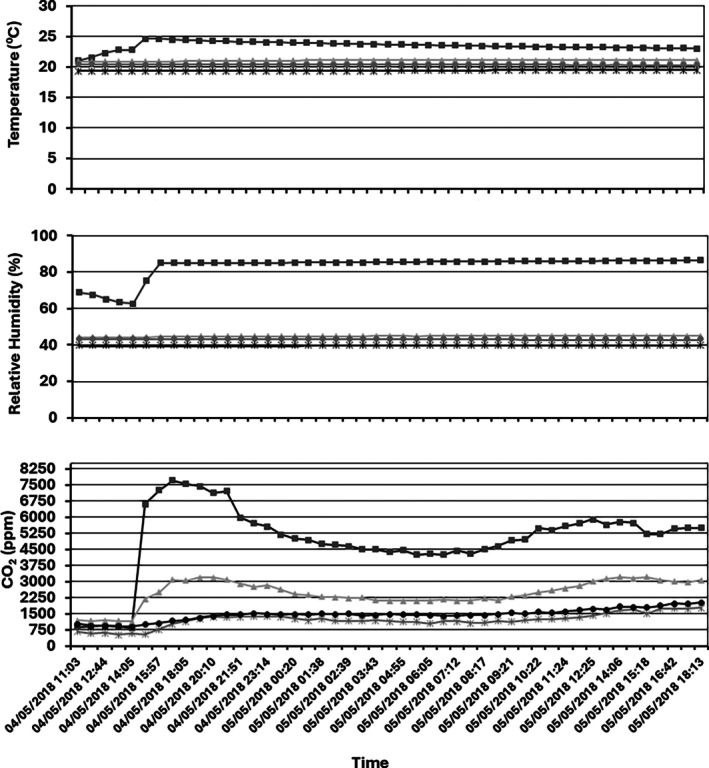
CO_2_ readings from the four nodes in cable 3 after increasing the m.c. in node 9 on 5 April 2018. Node 9 (square marker), node 10 (triangle marker), node 11 (circle marker) and node 12 (star marker) within an outside pilot in Barilla (Italy).

Figure 6 shows CO_2_ changes in the silo after creating the hotspot. Statistical analysis confirmed that CO_2_ concentrations were significantly higher in areas where mycotoxins were subsequently detected (Mann–Whitney *U* = 2847, *P* < 0.001). CO_2_ changes were observed throughout the silo, demonstrating that this variable can be monitored from areas spatially distant from the contamination source. The diffusion of CO_2_ throughout the silo appeared to be anisotropic – higher in the downward direction than in the horizontal direction.

Several mycotoxins were quantified in the wheat samples, indicating the likely presence of different fungal genera, including *Aspergillus*, *Penicillium* and *Fusarium*. Mycotoxin analysis of aggregate samples collected near and far from a CO₂ hotspot revealed clear differences in contamination levels (Table 1). In the low‐CO₂ area, all compounds were below the LOD or present at minimal concentrations. In contrast, samples from the high‐CO₂ hotspot showed significant contamination, including aflatoxin B1 (30.7 μg kg^−1^) and B2 (3.0 μg kg^−1^), both exceeding the EU legal limit (2.0 and 4.0 μg kg^−1^ for the sum of aflatoxins, respectively). Deoxynivalenol and its modified form deoxynivalenol‐3‐glucoside were also detected at 131.8 and 13.1 μg kg^−1^, respectively, along with moniliformin (26.8 μg kg^−1^), though all remained below existing regulatory thresholds. These findings suggest a spatial association between elevated CO₂ levels and increased mycotoxin accumulation, likely driven by localised fungal activity near the hotspot.

**Table 1:** Mycotoxin values quantified in aggregate samples from the area close to and far from the hotspot

Mycotoxin	Low‐CO_2_ area (μg kg^−1^)	High‐CO_2_ area hotspot (μg kg^−1^)	EU legal limit (μg kg^−1^)^a^
Aflatoxin B_1_	<LOD	30.7	2.0
Aflatoxin B_2_	<LOD	3.0	4.0 (sum of B_1_, B_2_, G_1_ and G_2_)
Ochratoxin A	<LOD	18.1	5.0
Deoxynivalenol	1	131.8	1250
Deoxynivalenol‐3‐glucoside	<LOD	13.1	No legal limit
Moniliformin	<LOD	26.8	No legal limit

^a^ Upper EU legal limit (EU, 1881/2006).

### Statistical analysis

Statistical analysis confirmed the relationship between elevated CO_2_ levels and mycotoxin contamination. CO_2_ concentrations were significantly higher in areas where mycotoxins were subsequently detected compared to areas with mycotoxin levels below detection limits (Mann–Whitney *U* = 2847, *P* < 0.001, *n*₁ = 1240, *n*₂ = 1680 measurements). The contaminated area (node 1) showed CO_2_ levels ranging from 800 to 10 000 ppm, while uncontaminated areas (nodes 2–4) remained within 400–3000 ppm.

Temporal analysis revealed significant increases in CO_2_ and RH parameters following the water addition event, while temperature changes were dominated by large‐scale environmental fluctuations that obscured detection of localised patterns. In the affected hotspot area (node 1), CO_2_ levels increased from a median of 1450 ppm (IQR: 1200–1680) pre‐water to 4200 ppm (IQR: 2800–6500) post‐water (*P* < 0.001), accompanied by a dramatic rise in relative humidity from baseline levels of 40–50% to saturation (100%) following water addition. In contrast, uncontaminated nodes (2–4) showed minimal CO_2_ elevation and maintained stable RH levels of 40–50% throughout the monitoring period.

Descriptive analysis revealed a clear threshold pattern: the node with CO_2_ peaks exceeding 6000 ppm corresponded to the area where mycotoxins exceeded EU legal limits, while all nodes maintaining CO_2_ levels below 3000 ppm showed mycotoxin concentrations below detection limits. Only the mycotoxin‐positive area showed RH saturation post‐water addition, while all other nodes remained stable at 40–50% RH.

The data suggest that CO_2_ thresholds above 3000 ppm, combined with localised RH increases above 80%, may serve as reliable indicators for mycotoxin risk, with sustained elevations above these levels preceding detectable mycotoxin formation by several days.

## DISCUSSION

This study examined the changes in respiration rates leading to CO_2_ production in natural stored wheat in both laboratory‐ and pilot‐scale silos. Laboratory experiments showed that dry wheat kernels (*a*_w_ = 0.80/16.5%) have a low respiration rate. However, significant increases in respiration were observed for wetter wheat at >18–21% m.c. (*a*_w_ = 0.88–0.90). Moisture content/temperature increases promote fungal growth and consequently the CO_2_ presence according to previous small‐scale laboratory studies with natural and artificially contaminated wheat with *F. graminearum* and *F. pseudograminearum*.^18^, ^19^ Also, kernels infected with filamentous fungal genera such as *Penicillium*, *Aspergillus* and *Fusarium* and a decrease of germination had been significantly correlated with the production of CO_2_.^12^, ^28^, ^29^, ^30^, ^31^

The statistical validation confirms that CO_2_ monitoring provides quantitative early warning of conditions conducive to mycotoxin formation. The significant elevation of CO_2_ levels in contaminated areas (*P* < 0.001) demonstrates the diagnostic potential of this approach, while the clear threshold patterns observed between contaminated and uncontaminated nodes support the development of alarm‐based monitoring systems.

*T*/HR/CO_2_ levels were monitored for 9 months in two pilot silos. Moreover, an artificial hotspot was created in one of the silos by increasing the m.c. of the kernels, aiming to simulate the effect of both a lack of tightness in the structure but also due to the effect of the decomposition of any animal commonly found in these environments (e.g. rodents, birds), which are common situations found at facilities during grain storage. Our results demonstrate that CO_2_ monitoring provides quantitative early warning of mycotoxin formation conditions. The significant elevation of CO_2_ levels in contaminated areas (*P* < 0.001) confirms the diagnostic potential of this approach and supports the development of alarm‐based monitoring systems with clear threshold values. Ileleji *et al*.^32^ and Maier *et al*.^33^ also reported that *T* alone might not be a reliable indicator of stored grain conditions in outdoor facilities, particularly in ground piles. It is worthwhile mentioning that changes in CO_2_ were detected even from areas far away for the initial hotspot and therefore fewer sensors may be required to monitor the whole silo. Our results showed that CO_2_ was an effective early indicator of fungal spoilage and mycotoxin accumulation. Portell *et al*.^34^ showed a positive correlation between CO_2_ and deoxynivalenol/zearalenone in artificially contaminated wheat by *F. graminearum* at *a*_w_ = 0.95 and 0.97.

Although mycobiota assessment was not performed in this study, quantified mycotoxin contamination (aflatoxins, ochratoxin A and trichothecenes) indicates that probably *Aspergillus*, *Penicillium* and *Fusarium* species were part of the natural mycobiota present in wheat. The presence of these three fungal genera and respective mycotoxins in stored wheat has been previously reported.^19^, ^35^, ^36^ High mycotoxin contamination was quantified in the artificially created hotspot after 6 days, indicating that remediation action such as grain aeration should be performed quickly to ensure food safety. Previously, Birck *et al*.^35^ reported CO_2_ diffusion efficiencies differ between spatial direction, particularly in horizontal warehouses containing maize or wheat. In these scenarios, CO_2_ diffusion was sensitively detected within 2 m of the hotspot in both the horizontal and downward directions as well as within 1 m of the hotspot in the upward direction. In addition, this may be a reliable parameter since no significant differences in the CO_2_ diffusion were found based on kernel orientation.^37^ Interestingly, Stone^31^ did not find any relationship between CO_*2*_ production and the presence of broken corn and foreign material. The spatial detection capability of CO_2_ monitoring demonstrated in this study offers significant advantages over traditional destructive sampling approaches. Kerry *et al*.^38^ investigated waste reduction through spatial analysis of grain bulks using destructive sampling methods, but highlighted the limitations of such approaches for continuous monitoring. In contrast, our CO_2_ sensor network provides non‐destructive, continuous spatial monitoring that can detect contamination hotspots without compromising grain integrity, offering a more sustainable approach to quality assessment. Previous research as well as results from our laboratory experiments (data not published) has shown that maize kernels have higher respiration rates compared to wheat as well as recently harvested kernels compared with the ones harvested in previous seasons.^37^

Previous experiments analysing response parameters of wheat/maize/peanut commodities (small scale of 10 g)^19^, ^20^, ^21^ as well as our current mini‐silo work were all carried out at Cranfield University. In previous experiments, gas chromatography was used to measure the temporal respiration rates while in this work two sets of ATEX‐compliant sensors were used at different scales due to the potential explosive atmosphere inside of the cereal silos. The first set of sensors used in the mini‐silos (1500 g) for a short period of time provided accurate readings (*T*/CO_2_), but CO_2_ sensors became easily saturated, reaching the maximum detection limit after 7 days at 17.5% m.c/25 °C and 15 days at 31% m.c/15 °C. Sensors tested in the pilot experiments (2 t) at Barilla (Italy) required some time for equilibration and needed recalibration after 1 year. Therefore, more engineering development is required to achieve accurate, accessible and long‐life technology for implementing standard operational procedures in silos. In addition, apart from the technological challenges there are still some challenges to the generation of models that can accurately predict risk and support decision making of silo managers. It is worthwhile mentioning that CO_2_ present in a silo is not exclusively generated by filamentous fungi, but it can also be produced by the cereals themselves and other pests present in the silo such as insects.

## CONCLUSIONS

Poor postharvest management can lead to rapid deterioration in grain quality, with severe decreases in germinability and nutritional value of the stored grain, possibly accompanied by undesirable fungal contamination and, consequently, mycotoxin production. Statistical validation confirms that CO_2_ monitoring provides quantitative early warning of mycotoxin formation conditions, with clear threshold patterns identified for risk assessment. CO_2_ changes were detected earlier than temperature and RH changes, with sustained elevations above 3000 ppm serving as reliable predictive indicators. Real‐time CO_2_ monitoring can therefore enable proactive grain management through threshold‐based alarm systems that trigger rapid remedial actions before visible spoilage occurs. In addition, sensors were able to detect CO_2_ changes from areas far away from the induced wet spot. Sensors used in this study showed some errant measurements and precise initial levels from 1 month after initial experimental setup. Therefore, there is a need to improve the technology and the affordability due to the current high cost of this technology. By analysing the time‐series data collected from the described silo setup, it is possible to develop prediction models for respiration rates. These models can then be used to inform decision support systems to aid in reducing food waste.

## AUTHOR CONTRIBUTIONS

Conceptualisation, NM, EG‐C and MS; methodology, EK, SZ, MSk, EG‐C; formal analysis, BI, MSk, EG‐C; writing – original draft preparation, EK, NM, EG‐C; review and editing, all the authors; project administration, NM, EG‐C; funding acquisition, NM. All authors have read and agreed to the published version of the manuscript.

## Supporting information


**Figure SA1.** Sensors next to the pilot silo inside and outside the company (Barilla, Italy).

**Figure SA2.** Temperature, relative humidity and carbon dioxide production monitored in four nodes of cable one inside the pilot silo (Barilla, Italy).

**Figure SA3:** Temperature, relative humidity and carbon dioxide production monitored in four nodes of cable one outside the pilot silo (Barilla, Italy).

## Data Availability

The data that support the findings of this study are available from the corresponding author upon reasonable request.

## References

[1] Alshannaq A and Yu JH , Occurrence, toxicity, and analysis of major mycotoxins in food. Int J Environ Res Public Health 14:632 (2017). 28608841 10.3390/ijerph14060632 PMC5486318

[2] Leslie JF , Moretti A , Mesterházy Á , Ameye M , Audenaert K , Singh PK *et al*., Key global actions for mycotoxin management in wheat and other small grains. Toxins 13:725 (2021). 34679018 10.3390/toxins13100725 PMC8541216

[3] García‐Cela E , Ramos AJ , Sanchis V and Marin S , Emerging risk management metrics in food safety: FSO, PO. How do they apply to the mycotoxin hazard? Food Control 25:797–808 (2012).

[4] European Commission, E. (2023). Commission Regulation (EU) 2023/915 of 25 April 2023 on maximum levels for certain contaminants in food and repealing Regulation (EC) No 1881/2006. Off J Eur Union 119:103–157 (2006).

[5] van Egmond HP , Schothorst RC and Jonker MA , Regulations relating to mycotoxins in food: perspectives in a global and European context. Anal Bioanal Chem 389:147–157 (2007). 17508207 10.1007/s00216-007-1317-9

[6] Olsen OA , Nuclear endosperm development in cereals and *Arabidopsis thaliana* . Plant Cell 16:214–227 (2004). 10.1105/tpc.017111 PMC2643391 15010513

[7] Magan N , Garcia‐Cela E , Verheecke‐Vaessen C and Medina A , Advances in post‐harvest detection and control of fungal contamination of cereals, in Advances in Postharvest Management of Cereals and Grains, 1st edn, ed. by Maier DE . Burleigh Dodds Science Publishing, Cambridge, pp. 265–287 (2020).

[8] Fleurat‐Lessard F , Integrated management of the risks of stored grain spoilage by seedborne fungi and contamination by storage mould mycotoxins – an update. J Stored Prod Res 71:22–40 (2017).

[9] Eskola M , Kos G , Elliott CT , Hajšlová J , Mayar S and Krska R , Worldwide contamination of food‐crops with mycotoxins: validity of the widely cited ‘FAO estimate’ of 25%. Crit Rev Food Sci Nutr 60:2773–2789 (2019). 31478403 10.1080/10408398.2019.1658570

[10] Kerry R , Ingram B , Garcia‐Cela E , Magan N , Ortiz BV and Scully B , Determining future aflatoxin contamination risk scenarios for corn in southern Georgia, USA using spatio‐temporal modelling and future climate simulations. Sci Rep 11:13522 (2021). 34188073 10.1038/s41598-021-92557-6 PMC8241871

[11] Sinha RN and Wallace HAH , Population dynamics of stored‐product mites. Oecologia 12:315–327 (1973). 28308234 10.1007/BF00345046

[12] Uddin MS , Armstrong PR and Zhang N , Accuracy of grain moisture content prediction using temperature and relative humidity sensors. Appl Eng Agric 22:267–273 (2006).

[13] Bhat C , Maier DE and Ileleji KE , Exploratory Use of a Portable CO_2_ Sensor for Early Detection of Spoilage in a Large Maize Storage Tank. St Joseph, Michigan, USA (2003).

[14] Maier DE , Channaiah LH , Martinez‐Kawas A , Lawrence JS , Chaves EV , Coradi PC *et al*., Monitoring carbon dioxide concentration for early detection of spoilage in stored grain. Julius‐Kuhn‐Arch 425:505 (2010).

[15] Ramachandran RP , Integrated approach on stored grain quality management with CO_2_ monitoring: a review. J Stored Prod Res. 96:101950 (2022).

[16] Danao MGC , Zandonadi RS and Gates RS , Development of a grain monitoring probe to measure temperature, relative humidity, carbon dioxide levels and logistical information during handling and transportation of soybeans. Comput Electron Agric 119:74–82 (2015).

[17] Nunes CF , Coradi PC and Jaques LBA , Sensor‐cable‐probe and sampler for early detection and prediction of dry matter loss and real‐time corn grain quality in transport and storage. Sci Rep 13:5686 (2023). 37029273 10.1038/s41598-023-32684-4 PMC10082028

[18] Cu H , Wang S , Yang X , Zhang W , Chen M , Wu Y *et al*., Predictive models for assessing the risk of *Fusarium pseudograminearum* mycotoxin contamination in post‐harvest wheat with multi‐parameter integrated sensors. Food Chem X 16 (2022). 10.1016/j.fochx.2022.100472 PMC9593717 36304207

[19] Garcia‐Cela E , Kiaitsi E , Sulyok M , Medina A and Magan N , *Fusarium graminearum* in stored wheat: use of CO_2_ production to quantify dry matter losses and relate this to relative risks of zearalenone contamination under interacting environmental conditions. Toxins (Basel) 10:86 (2018). 29462982 10.3390/toxins10020086 PMC5848187

[20] Garcia‐Cela E , Kiaitsi E , Sulyok M , Krska R , Medina A , Petit Damico I *et al*., Influence of storage environment on maize grain: CO_2_ production, dry matter losses and aflatoxins contamination. Food Addit Contam Part A 36:175–185 (2019). 10.1080/19440049.2018.1556403 30638440

[21] Garcia‐Cela E , Sanchez FG , Sulyok M , Verheecke‐Vaessen C , Medina A , Krska R *et al*., Carbon dioxide production as an indicator of *Aspergillus flavus* colonisation and aflatoxins/cyclopiazonic acid contamination in shelled peanuts stored under different interacting abiotic factors. Fungal Biol 124:1–7 (2020). 31892372 10.1016/j.funbio.2019.10.003

[22] Martín Castaño S , Medina A and Magan N , Impact of storage environment on respiration, dry matter losses and fumonisin B1 contamination of stored paddy and brown rice. World Mycotoxin J 10:319–326 (2017).

[23] Martín Castaño S , Medina A and Magan N , Comparison of dry matter losses and aflatoxin B1 contamination of paddy and brown rice stored naturally or after inoculation with *Aspergillus flavus* at different environmental conditions. J Stored Prod Res. 73:47–53 (2017).

[24] Mylona K and Magan N , *Fusarium langsethiae*: storage environment influences dry matter losses and T2 and HT‐2 toxin contamination of oats. J Stored Prod Res 47:321–327 (2011).

[25] Mylona K , Sulyok M and Magan N , Relationship between environmental factors, dry matter loss and mycotoxin levels in stored wheat and maize infected with *Fusarium* species. Food Addit Contam Part A 29:1118–1128 (2012). 10.1080/19440049.2012.672340 22494580

[26] Ingram B , Marin S , Kiaitsi E , Magan N , Verheecke‐Vaessen C , Cervini C *et al*., *Fusarium graminearum* and zearalenone in wheat: a water activity–temperature model. Fungal Biol 129:101572 (2025). 40441792 10.1016/j.funbio.2025.101572

[27] Sulyok M , Stadler D , Steiner D and Krska R , Validation of an LC‐MS/MS‐based dilute‐and‐shoot approach for the quantification of >500 mycotoxins and other secondary metabolites in food crops: challenges and solutions. Anal Bioanal Chem 412:2607–2620 (2020). 32078002 10.1007/s00216-020-02489-9 PMC7136310

[28] White NDG , Sinha RN and Muir WE , Intergranular carbon dioxide as an indicator of biological activity associated with the spoilage of stored wheat. Cereal Bulk Eng J 24:35–42 (1982).

[29] Singh D , Muir WE and Sinha RN , Apparent coefficient of diffusion of carbon dioxide through samples of cereals and rapeseed. J Stored Prod Res 20:169–175 (1984).

[30] Fernandez A , Stroshine R and Tuite J , Mold growth and carbon dioxide production during storage of high‐moisture corn. Cereal Chem 62:137–143 (1985).

[31] Sone J , Carbon dioxide production in stored maize as affected by moisture content, level of broken corn and foreign materials and infestation by *Sitophilus zeamais* Motschulsky. J Asia Pac Entomol 2:133–141 (1999).

[32] Ileleji KE , Maier DE , Bhat C and Woloshuk CP , Detection of a developing hot spot in stored corn with a CO_2_ sensor. Appl Eng Agric 22:275–289 (2006).

[33] Maier DE , Hulasare R , Qian B and Armstrong PR , Monitoring carbon dioxide levels for early detection of spoilage and pests in stored grain. 9th Int Working Conf Stored Prod Prot (2006).

[34] Portell X , Verheecke‐Vaessen C , Torrelles‐Ràfales R , Medina A , Otten W , Magan N *et al*., Three‐dimensional study of *F. graminearum* colonisation of stored wheat: post‐harvest growth patterns, dry matter losses and mycotoxin contamination. Microorganisms 8:1170 (2020). 32752221 10.3390/microorganisms8081170 PMC7465026

[35] Birck NMM , Lorini I and Scussel VM , Fungus and mycotoxins in wheat grain at post harvest. 9th Int Working Conf Stored Prod Prot:198–205 (2006).

[36] Joshaghani H , Namjoo M , Rostami M , Kohsar F and Niknejad F , Mycoflora of fungal contamination in wheat storage (silos) in Golestan Province, north of Iran. Jundishapur J Microbiol 6 (2013).

[37] Shunmugam G , Jayas DS , White NDG and Muir WE , Diffusion of carbon dioxide through grain bulks. J Stored Prod Res 41:131–144 (2005).

[38] Kerry R , Ingram B , Garcia‐Cela E and Magan N , Investigation of the potential to reduce grain waste through sampling and spatial analysis of grain bulks. Biosyst Eng 207:92–105 (2021).

