# Supplementary figures and images for: Real‐time CO2 monitoring for early detection of grain spoilage and mycotoxin contamination
Source: J Sci Food Agric. 2025 Aug 28;105(15):8985–93. doi: 10.1002/jsfa.70151 (PMC12595400; doi:10.1002/jsfa.70151)

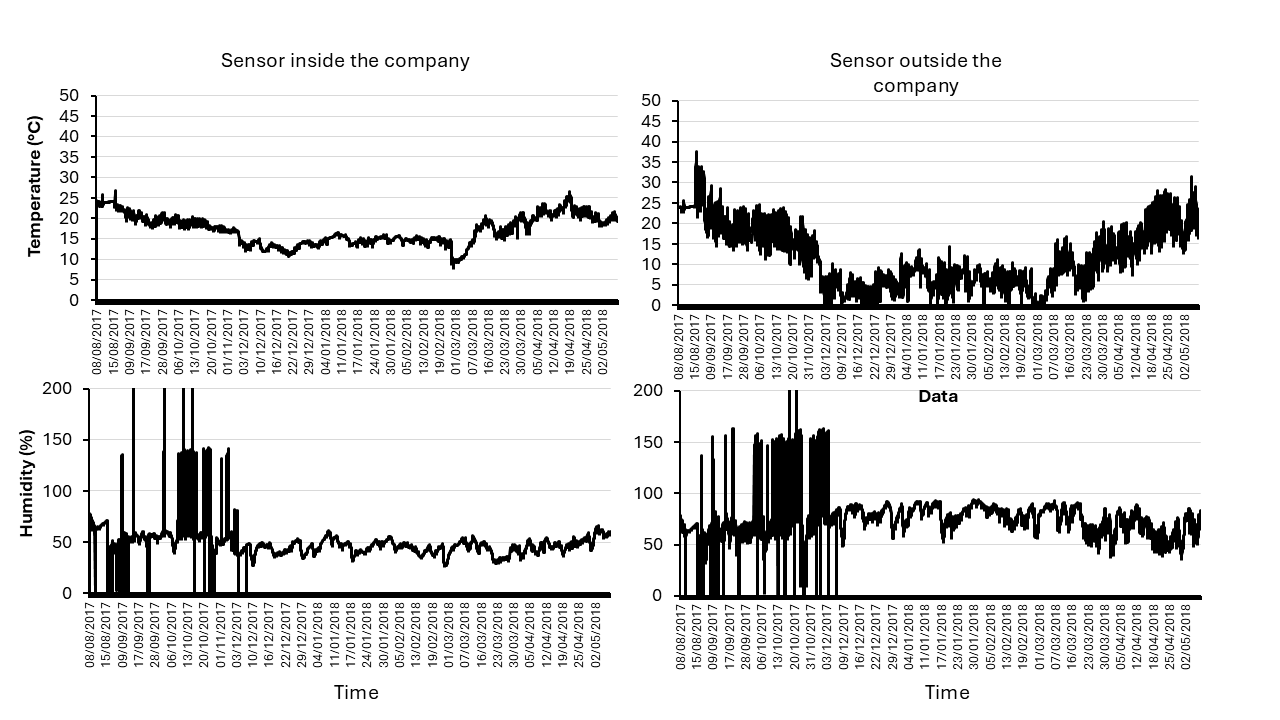

Supplement: Supplementary file 1 — Figure SA1. Sensors next to the pilot silo inside and outside the company (Barilla, Italy). [file JSFA-105-8985-s001.tif]

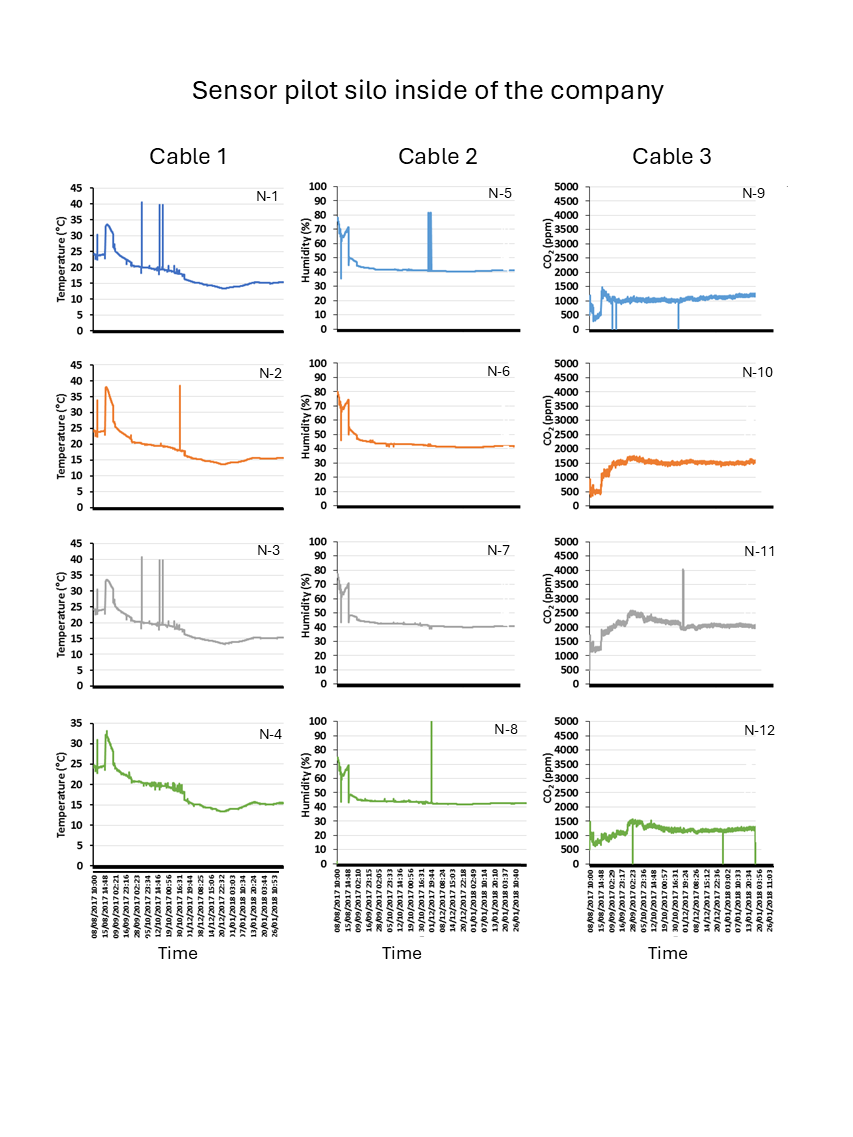

Supplement: Supplementary file 2 — Figure SA2. Temperature, relative humidity and carbon dioxide production monitored in four nodes of cable one inside the pilot silo (Barilla, Italy). [file JSFA-105-8985-s003.tif]

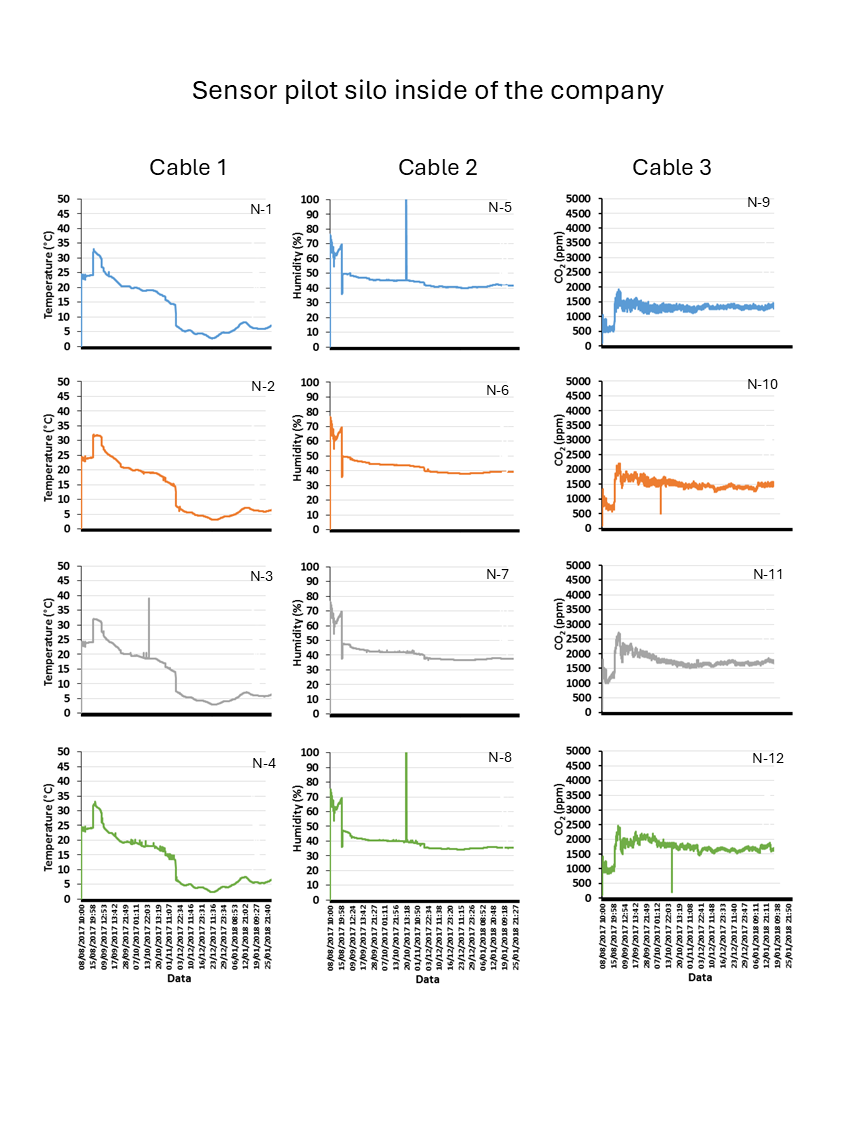

Supplement: Supplementary file 3 — Figure SA3: Temperature, relative humidity and carbon dioxide production monitored in four nodes of cable one outside the pilot silo (Barilla, Italy). [file JSFA-105-8985-s002.tif]
